# Effectiveness of a Web 2.0 Intervention to Increase Physical Activity in Real-World Settings: Randomized Ecological Trial

**DOI:** 10.2196/jmir.8484

**Published:** 2017-11-13

**Authors:** Corneel Vandelanotte, Gregory S Kolt, Cristina M Caperchione, Trevor N Savage, Richard R Rosenkranz, Anthony J Maeder, Anetta Van Itallie, Rhys Tague, Christopher Oldmeadow, W Kerry Mummery, Mitch J Duncan

**Affiliations:** ^1^ Central Queensland University Rockhampton Australia; ^2^ Western Sydney University Sydney Australia; ^3^ University of British Columbia Kelowna, BC Canada; ^4^ School of Allied Health Sciences Griffith University Southport Australia; ^5^ Kansas State University Manhattan, KS United States; ^6^ Flinders University Adelaide Australia; ^7^ University of Newcastle Newcastle Australia; ^8^ University of Alberta Edmonton, AB Canada

**Keywords:** Internet, online, Web based, behavioral intervention, external validity, pragmatic trial

## Abstract

**Background:**

The translation of Web-based physical activity intervention research into the real world is lacking and becoming increasingly important.

**Objective:**

To compare usage and effectiveness, in real-world settings, of a traditional Web 1.0 Web-based physical activity intervention, providing limited interactivity, to a Web 2.0 Web-based physical activity intervention that includes interactive features, such as social networking (ie, status updates, online “friends,” and personalized profile pages), blogs, and Google Maps mash-ups.

**Methods:**

Adults spontaneously signing up for the freely available 10,000 Steps website were randomized to the 10,000 Steps website (Web 1.0) or the newly developed WALK 2.0 website (Web 2.0). Physical activity (Active Australia Survey), quality of life (RAND 36), and body mass index (BMI) were assessed at baseline, 3 months, and 12 months. Website usage was measured continuously. Analyses of covariance were used to assess change over time in continuous outcome measures. Multiple imputation was used to deal with missing data.

**Results:**

A total of 1328 participants completed baseline assessments. Only 3-month outcomes (224 completers) were analyzed due to high attrition at 12 months (77 completers). Web 2.0 group participants increased physical activity by 92.8 minutes per week more than those in the Web 1.0 group (95% CI 28.8-156.8; P=.005); their BMI values also decreased more (–1.03 kg/m2, 95% CI –1.65 to -0.41; P=.001). For quality of life, only the physical functioning domain score significantly improved more in the Web 2.0 group (3.6, 95% CI 1.7-5.5; P<.001). The time between the first and last visit to the website (3.57 vs 2.22 weeks; P<.001) and the mean number of days the website was visited (9.02 vs 5.71 days; P=.002) were significantly greater in the Web 2.0 group compared to the Web 1.0 group. The difference in time-to-nonusage attrition was not statistically significant between groups (Hazard Ratio=0.97, 95% CI 0.86-1.09; P=.59). Only 21.99% (292/1328) of participants (n=292 summed for both groups) were still using either website after 2 weeks and 6.55% (87/1328) were using either website after 10 weeks.

**Conclusions:**

The website that provided more interactive and social features was more effective in improving physical activity in real-world conditions. While the Web 2.0 website was visited significantly more, both groups nevertheless displayed high nonusage attrition and low intervention engagement. More research is needed to examine the external validity and generalizability of Web-based physical activity interventions.

**Trial Registration:**

Australian New Zealand Clinical Trials Registry: ACTRN12611000253909; https://anzctr.org.au /Trial/Registration/TrialReview.aspx?id=336588&isReview=true (Archived by WebCite at http://www.webcitation.org/6ufzw 2HxD)

## Introduction

Given low population levels of physical activity and high associated physical and mental burden of disease caused by inactive lifestyles [1], there is a need for effective physical activity interventions that can reach large populations at low cost [2]. In this context, research into the effectiveness of Web-based interventions has become popular [3], as large and increasingly diverse populations can be reached without geographical limitations by using the Internet [4]. While literature reviews and meta-analyses point to the short-term effectiveness of Web-based physical activity interventions [5,6], they also highlight that there is a lack of evidence for long-term behavior change, as well as a lack of knowledge about what are the most effective intervention components [7]. The lack of evidence for long-term behavior change has often been attributed to low levels of participant engagement and retention, due to examining websites that are static in nature, that lack social support elements, and that provide limited opportunity for interactivity or information exchange [5].

Websites with more dynamic, interactive, user-focused features, also referred to as *second generation* or *Web 2.0* features, are now commonly used and include social networking, blogs, wikis, podcasts, and mash-ups [8]. They provide users with the opportunity to directly generate, modify, and share information [9]. Few physical activity studies have examined the effectiveness of Web 2.0 features [8,10,11], which may enhance engagement with the intervention and, in turn, lead to long-term behavior change. A review by Maher et al, however, indicated that the use of online social networks in behavior change trials was only modestly effective [10]. In this context, it has been argued that randomized controlled trials (RCTs) are not the most appropriate research design to truly examine the effectiveness of Web 2.0 features [12].

It has been asserted that the highly controlled nature of RCTs, which aim to minimize impact of selection bias, confounding factors, and contamination, stifle the dynamic, spontaneous, viral nature of Web 2.0 features [12]. While RCTs are an essential component of the research process, complementary approaches with high external validity and generalizability are also essential. For example, if one is not able to invite friends to join an online social network due to RCT-related restrictions, the social network is unlikely to be as functional and effective as it would be in real-world circumstances [13]. As such, there is a need for alternative and ecologically valid research designs that evaluate Web-based interventions in real-life conditions in order to advance the science in this area [14].

The translation and dissemination of Web-based physical activity intervention research into the real world is lacking and becoming increasingly important. Therefore, the main aim of this study was to compare the physical activity behavior of individuals using a traditional Web 1.0 physical activity website to those using an innovative Web 2.0 physical activity website in real-world settings. The study also aimed to assess the effectiveness of Web 2.0 features to engage and retain individuals to a physical activity promotion website, as well as examine differences in quality of life and body mass index (BMI) between intervention groups. The primary hypothesis was that participants in the Web 2.0 condition would display higher levels of physical activity at 3 and 12 months, compared to those in the Web 1.0 condition. The secondary hypotheses were that, in the Web 2.0 condition, there would be higher website engagement and retention as well as improvements in quality of life and BMI when compared to the Web 1.0 condition at 3 and 12 months.

## Methods

### Overview

As the detailed protocol for this study has been published elsewhere [13], only summary information will be provided here (see Multimedia Appendix 1 for additional screenshots of the intervention). This study is the second phase of the substantive WALK 2.0 project and builds on an earlier RCT [15,16], which rigorously tested the efficacy of the interventions described here [11]. The Western Sydney University Human Research Ethics Committee granted ethical approval for this study (H8767). This trial has been registered at the Australian New Zealand Clinical Trials Registry (ACTRN12611000253909).

### Recruitment, Procedures, Study Design, and Participants

Adults 18 years of age or older spontaneously signing up for the freely available and Web-based 10,000 Steps program [17], which attracts over a 1000 new members per month [18], were asked during the registration process whether they wanted to participate in a research study from November 2012 to June 2014. The 10,000 Steps project has been funded since 2001 through Queensland Health, one of the Australian State Ministries of Health. The project is well known through media and marketing events in Australia, particularly in Queensland, with over 70% program awareness in population-based surveys. If potential participants agreed to participate in research, they received more information about the study, were screened online for eligibility, provided informed consent, and completed a brief baseline survey. Using a computer-generated algorithm, they were randomized to receive access to one of two intervention websites: a Web 1.0 intervention, which was the 10,000 Steps website they were originally signing up for, or a Web 2.0 intervention, which was the newly developed WALK 2.0 website. For technical reasons, participants were randomized before completing the baseline measures, however, they only gained access to intervention materials after completing the baseline assessment. Follow-up outcomes were assessed 3 and 12 months postbaseline using online questionnaires; participants were invited by email and received up to three reminders. All actions, from study invitation to completion, were fully automated with no interaction from the research team at any point. The research team also provided no instructions as to how the interventions should be used and there was no predefined intervention duration. However, even though the aim of reaching 10,000 steps a day was implicit, participants were provided with the Australian Physical Activity Guidelines and the websites were designed to encourage self-monitoring and interaction on a daily basis for as long as possible—participants had an option to receive a daily reminder to use the websites. No pedometers were provided. Exclusion criteria were the following: being under 18 years of age; seeking to participate in a 10,000 Steps Workplace Challenge; having been a participant in the WALK 2.0 RCT; and having a medical condition that prevents them from increasing physical activity, assessed through the Physical Activity Readiness Questionnaire [19].

### Interventions

#### Web 1.0 Intervention

Participants allocated to the Web 1.0 group were given access to the existing 10,000 Steps website. This website was originally developed to promote the community-based 10,000 Steps Australia project [20,21]. The website includes features that support individual self-monitoring (eg, step log) and communication exchange (eg, discussion forums), and provides access to a library with educational resources (eg, benefits of activity). Participants were able to log steps and/or type and duration of other physical activities. Participants also had the ability to share stories, ask questions, or make comments in the discussion forum.

#### Web 2.0 Intervention

Participants allocated to the Web 2.0 group were given access to a newly developed website, WALK 2.0, that provided content and functionality similar to the Web 1.0 condition (eg, step log and library); however, this website was supplemented with Web 2.0 features that included annotation, messaging, and group-publishing tools implemented in a stand-alone social networking setting. Participants in the Web 2.0 group also had access to self-monitoring features and educational resources, however, these had advanced functionalities that provided greater interactivity and participatory communication between users (eg, status updates, internal emails, inviting “friends,” and personalized profile pages). Participants could upload content to their own profile page, share this information with others, and invite individuals who were not study participants to become their “friends” and use the website. Participants could also connect with Facebook (eg, post their step total for the day on their Facebook wall), but the Web 2.0 website was completely separate from Facebook.

### Measures

#### Demographics

Participants’ gender, age, educational level (school education, trade/diploma, or higher education), employment status (full time, part time/casual, or other), occupation (professional white collar, blue collar, or other), weekly household income (<Aus $1000, Aus $1000-$1999, Aus $2000-$5000, or no response), Internet self-confidence (low or high), height (cm), and weight (kg) were assessed. Self-reported BMI was calculated as weight (kg) over height squared (m^2^) and categorized as normal weight (≤24.99 kg/m^2^), overweight (25-29.99 kg/m^2^), and obese (≥30 kg/m^2^).

#### Physical Activity

The Active Australia Survey was used to measure self-reported physical activity [22]. This instrument provides an estimate of total weekly minutes of physical activity by summing total minutes of walking for transport and recreation, moderate-intensity physical activity, and vigorous-intensity physical activity—multiplied by 2 to account for the higher energy expenditure per time unit—during the previous week. The Active Australia Survey has acceptable test-retest reliability and validity in Australian adults [23,24] and has been demonstrated to be sensitive enough to detect change over time [25]. The Active Australia Survey was used to determine the following: total physical activity per week and whether participants were engaging in sufficient physical activity—a minimum of 150 minutes of moderate-to-vigorous physical activity per week accumulated over five or more sessions.

#### Quality of Life

The RAND 36 Short Form Survey was used to assess quality of life in eight health-related categories: physical functioning, bodily pain, role limitations due to physical health problems, role limitations due to personal or emotional problems, emotional well-being, social functioning, energy/fatigue, and general health perceptions [26,27]. All items were scored from 0 to 100, with a high score representing a more favorable health state. Items in each category were then averaged together to create eight subscale scores. The RAND 36 has been validated in Australian populations [28].

#### Website Engagement and Retention

Website usage statistics for both websites were continuously measured using Google Analytics (eg, time on site) and data were extracted directly from the website databases (eg, step entry information). These measures were only examined from baseline to 3 months (first 12 weeks), due to the low survey completion rate at 12 months. The total and average number of website visits were assessed, as well as the time between the first and last visit. The total and average number of days with a step entry and step entry comments were assessed, including the time between first and last step entry. Nonusage attrition was defined as not having visited the website and/or logged steps for at least two consecutive weeks [11,18].

#### Website Usability

The System Usability Scale (SUS) was used to assess website usability [29]. This scale is a 10-item survey, scored on a 5-point scale of strength of agreement, with good reliability and concurrent validity [30]. Final scores can range from 0 to 100, where higher scores indicate better usability. Self-reported use and usefulness for different features on both websites is also reported. Participants were asked about usefulness on a 5-point scale; the proportion of participants who thought the feature was “useful” or “very useful” is reported.

### Sample Size

 The trial was powered to detect a 4% between-group difference in the prevalence of sufficient physical activity, as defined by the Australian Physical Activity Guidelines, between the Web 1.0 and Web 2.0 groups. To achieve this aim with 80% power and an alpha level of .05, a minimum of 1034 participants per group were needed [13].

### Statistical Analyses

All statistical analyses were conducted using SAS version 9.4 (SAS Institute Inc). Differences between participants with complete and missing data were compared using *t* tests or Pearson chi-square tests. Analysis of covariance (ANCOVA) was used to test for differences between treatment groups at 3 months in physical activity, quality of life, and BMI; baseline physical activity, BMI, quality of life levels, and confounding variables were included in the models as covariates. Logistic regression was used to estimate between-group differences in the proportion of participants who achieved sufficient physical activity. Results are presented both for those with complete data at baseline and 3 months (*completer analyses*), as well as those with missing data, following intention-to-treat principles. Multiple imputation was applied to deal with missing data, under the missing-at-random assumption, using the chained equations method. Rubin’s method was used to pool the treatment effects using 25 imputed datasets, as the fraction of missing data was high [31]. To analyze between-group differences in website engagement and retention, *t* tests were used. A proportional hazards regression model was used to estimate between-group differences in time from randomization to nonusage; Kaplan-Meier estimates of the proportion remaining active (the *survival distribution*) are also presented [32]. Due to very small participant numbers, 12-month data were not included in any of the analyses. The significance level was set at *P*<.05.

## Results

### Participants

**Figure 1:**
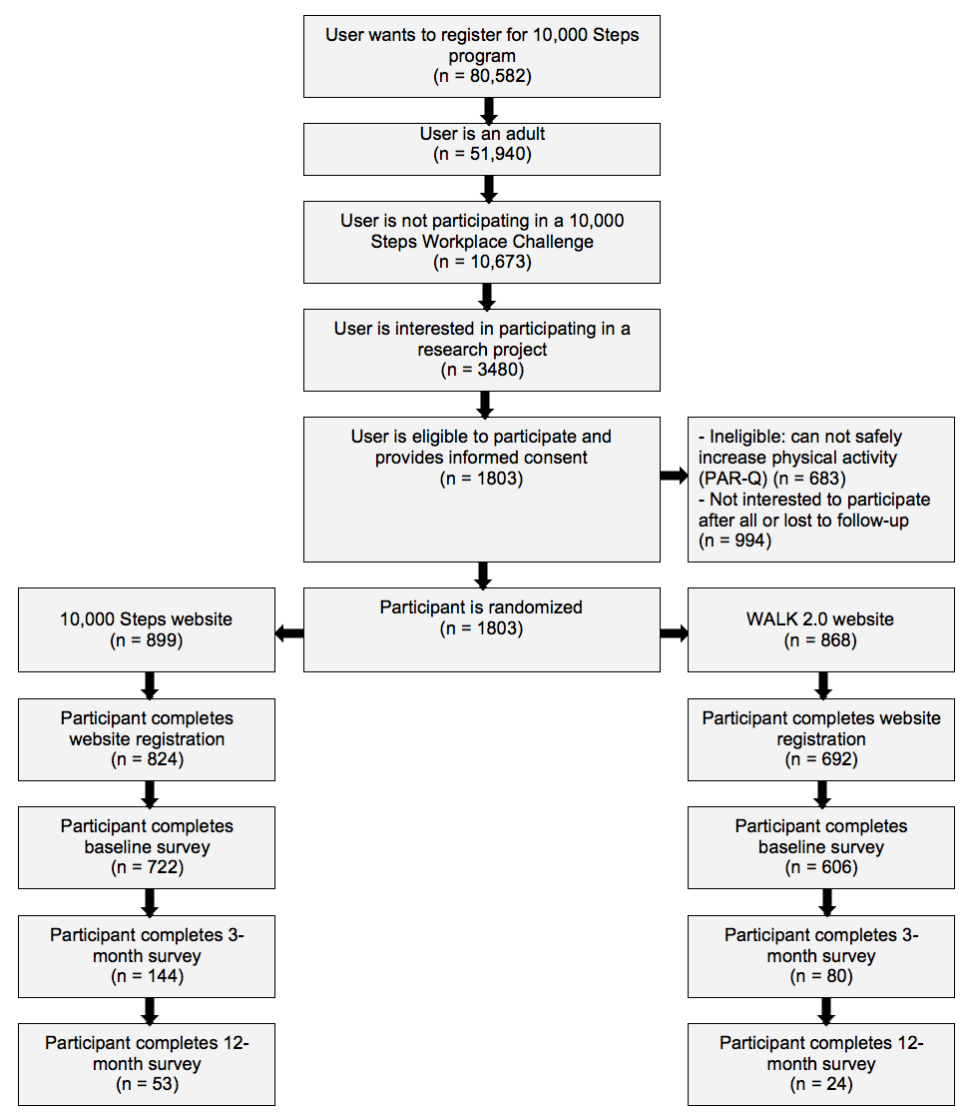
Participant flowchart. PAR-Q: Physical Activity Readiness Questionnaire.

A participant flowchart is provided in Figure 1. After automatically screening out ineligible people, 10,673 people were invited and 3480 indicated an interest in participating. After eligibility checks, providing informed consent, and website registration, 1328 people completed all baseline measures. Out of 1328 participants, 224 (16.87%) completed the 3-month assessment and 77 (5.80%) completed the 12-month assessment. Table 1 presents participant demographics. At baseline, the majority of participants were female (1095/1328, 82.45%), were 44 years of age or under (818/1328, 61.60%), were overweight or obese (849/1328, 63.93%), had a higher education (693/1328, 52.18%), were full-time employed (771/1328, 58.06%), had a professional or white-collar job (879/1328, 66.19%), and participated in sufficient physical activity (757/1328, 57.00%). There were no significant between-group differences in participant characteristics at baseline; however, several between-group differences were observed among those who completed the 3-month assessment and those who did not. There was significantly greater retention among those who were Web 1.0 group participants, male, aged 45 years or older, and not obese, as well as those having a higher education, professional occupation, and higher income.

### Physical Activity

The physical activity outcomes are presented in Table 2. In the intention-to-treat analysis, a significant difference between groups was found: participants randomized to the Web 2.0 group increased physical activity by 92.8 minutes per week more compared to those in the Web 1.0 group (*P*=.005; Cohen *d* effect size=0.29). In the completer analysis, participants in the Web 2.0 group increased physical activity by 56.6 minutes per week more compared to those in the Web 1.0 group, however, this difference was not significant (*P*=.20; Cohen *d* effect size=0.24).

At baseline, 57.00% (757/1328) of participants in both groups engaged in sufficient physical activity. At the 3-month time point, 77% (62/80) of Web 2.0 participants and 71.5% (103/144) of Web 1.0 participants engaged in sufficient physical activity. A significant between-group difference in favor of the Web 2.0 group was observed in the intention-to-treat analysis (Relative Risk [RR]=1.11, 95% CI 1.01-1.21; *t*_64.5_=2.19, *P*=.03), but not in the completer analysis (RR=1.03, 95% CI 0.89-1.18; Z=0.36, *P*=.71).

### Quality of Life

There was no effect of the intervention on most quality of life variables (see Table 2), except for the physical functioning domain: a significant improvement (3.6 units) was observed in the Web 2.0 group compared to the Web 1.0 group (*P*<.001).

### Body Mass Index

BMI reduced over time in both groups (see Table 2) and significant between-group differences were observed for both the intention-to-treat analysis (in favor of the Web 2.0 group: change in BMI=-1.03 kg/m^2^, *P*=.002) and the completer analysis (in favor of the Web 1.0 group: change in BMI=-0.58 kg/m^2^, *P*=.002).

**Table 1:** Participant demographic characteristics at baseline by group and for those with complete or missing data.

Demographic characteristics		Total (n=1328)	Web 1.0: 10,000 Steps (n=722)	Web 2.0: WALK 2.0 (n=606)	Completer at 3 months (n=224)	Missing at 3 months (n=1104)	*P* value^a^
**Group, n (%)**							
	10,000 Steps	722 (54.36)	722 (100)	0 (0)	144 (64.3)	578 (52.35)	.001
	WALK 2.0	606 (45.63)	0 (0)	606 (100)	80 (35.7)	526 (47.73)	
**Gender, n (%)**							
	Male	233 (17.54)	117 (16.2)	116 (19.1)	54 (24.1)	179 (16.21)	.006
	Female	1095 (82.45)	605 (83.8)	490 (80.9)	170 (75.9)	925 (83.78)	
**Age (years), n (%)**							
	18-34	503 (37.87)	278 (38.5)	225 (42.1)	52 (23.2)	451 (40.85)	<.001
	35-44	315 (23.71)	163 (23.6)	152 (25.1)	45 (20.1)	270 (24.45)	
	45-54	310 (23.34)	174 (24.1)	136 (22.4)	67 (29.9)	243 (22.01)	
	55-64	164 (12.34)	83 (11.5)	81 (13.4)	43 (19.2)	121 (10.96)	
	65 and over	36 (2.71)	24 (3.3)	12 (2.0)	17 (7.6)	19 (1.72)	
**Internet self-confidence, n (%)**							
	Low	38 (2.86)	24 (3.3)	14 (2.3)	6 (2.7)	32 (2.89)	.86
	High	1289 (97.06)	698 (96.7)	591 (97.5)	218 (97.3)	1071 (97.01)	
**Body mass index (kg/m^2^), n (%)**							
	Normal weight (≤24.99)	438 (32.98)	239 (33.1)	199 (32.8)	83 (37.1)	355 (32.15)	.008
	Overweight (25-29.99)	376 (28.31)	202 (28.0)	174 (23.8)	75 (33.5)	301 (27.26)	
	Obese (≥30)	473 (35.61)	259 (35.9)	214 (35.3)	61 (27.2)	412 (37.31)	
**Highest education level, n (%)**							
	Higher education	698 (51.88)	362 (50.1)	336 (55.4)	135 (60.3)	563 (50.99)	.03
	Trade/diploma	428 (32.22)	240 (33.2)	188 (31.0)	64 (28.6)	364 (32.97)	
	School education	201 (15.13)	120 (16.6)	81 (13.4)	25 (11.2)	176 (15.94)	
**Employment, n (%)**							
	Full time	771 (58.05)	425 (58.9)	346 (57.1)	140 (61.9)	631 (57.15)	.35
	Part time/casual	263 (19.80)	130 (18.0)	133 (21.9)	39 (17.4)	224 (20.28)	
	Other	293 (22.06)	167 (23.1)	126 (20.8)	45 (20.1)	248 (22.46)	
**Occupation, n (%)**							
	Professional	540 (40.66)	298 (40.0)	242 (39.9)	111 (49.6)	429 (38.85)	.04
	White collar	339 (25.52)	177 (24.5)	162 (26.7)	45 (20.1)	294 (26.63)	
	Blue collar	37 (2.78)	19 (2.6)	18 (3.0)	7 (3.1)	30 (2.71)	
	Other	119 (8.96)	61 (8.4)	58 (9.6)	16 (7.1)	103 (9.32)	
	No response	293 (22.06)	167 (23.1)	126 (20.8)	45 (20.1)	248 (22.46)	
**Weekly household income (Aus $), n (%)**							
	<$1000	361 (27.18)	193 (26.7)	168 (27.7)	40 (18.3)	321 (29.07)	.001
	$1000-$1999	372 (28.01)	207 (28.7)	165 (27.2)	74 (33.0)	298 (26.99)	
	$2000-$5000	324 (24.39)	173 (24.0)	151 (24.9)	67 (29.9)	257 (23.27)	
	No response	270 (20.33)	149 (20.6)	121 (20.0)	43 (19.2)	227 (20.56)	
Total weekly minutes of physical activity, mean (SD)		338 (348)	341 (353)	334 (342)	306 (270)	344 (361)	.13
							
							
**Level of physical activity, n (%)**							
	Sufficient	757 (57.00)	410 (56.8)	347 (57.3)	141 (62.9)	616 (55.79)	.13
	Insufficient	496 (37.34)	274 (38.0)	222 (36.6)	71 (32.0)	425 (38.49)	
	No reported activity	75 (5.64)	38 (5.3)	37 (6.1)	12 (5.4)	63 (5.70)	

^a^*P* values compare those who completed or were missing at the 3-month time point. Statistical significance is represented by *P*<.05.

**Table 2:** Changes in outcome variables for intention-to-treat (n=1328) and completer (n=224) analyses.

Outcome variables			Web 1.0: 10,000 Steps		Web 2.0: WALK 2.0		Difference in between- group changes (95% CI)	Z or *t*^a^	*P* value^b^
			Baseline, mean (SD)	3 months, mean (SD/SE^c^)	Baseline, mean (SD)	3 months, mean (SD/SE^c^)			
**Total physical activity (min/week)**^d^									
		Intention-to-treat	341.9 (353.8)	381.7 (16.6)	334.0 (342.2)	473.9 (26.4)	+92.78 (28.78- 156.77)	2.91	.005
		Completers	341.9 (353.8)	385.2 (269.5)	334.0 (342.2)	464.5 (359.0)	+56.65 (-30.35-143.64)	1.28	.20
**Quality of life**^e^									
	**Physical functioning**								
		Intention-to-treat	88.8 (15.5)	89.1 (0.8)	88.5 (16.2)	92.7 (0.5)	+3.58 (1.66-5.49)	3.72	˂.001
		Completers	88.8 (15.5)	89.6 (14.5)	88.5 (16.2)	92.9 (7.7)	+3.79 (0.68-6.91)	2.39	.02
	**Role limitations due to physical health problems**								
		Intention-to-treat	83.8 (29.3)	83.8 (1.6)	84.2 (29.0)	84.2 (1.7)	+0.38 (-4.85-5.62)	0.15	.88
		Completers	83.8 (29.3)	84.2 (29.0)	84.2 (29.0)	85.3 (28.0)	-0.14 (-8.18-7.90)	0.04	.97
	**Role limitation due to personal or emotional problems**								
		Intention-to-treat	74.3 (36.9)	80.6 (2.1)	76.2 (36.8)	84.0 (1.8)	+3.31 (-2.59-9.22)	1.12	.27
		Completers	74.3 (36.9)	80.1 (35.4)	76.2 (36.8)	85.0 (28.8)	+5.83 (-2.68-14.34)	1.34	.18
	**Energy/fatigue**								
		Intention-to-treat	50.0 (20.7)	57.2 (1.1)	51.4 (20.4)	59.5 (1.4)	+2.28 (-1.49-6.06)	1.21	.23
		Completers	50.0 (20.7)	57.8 (21.2)	51.4 (20.4)	60.2 (19.0)	+1.55 (-2.84-5.94)	0.69	.49
	**Emotional well-being**								
		Intention-to-treat	69.3 (19.2)	76.4 (0.9)	70.2 (19.1)	78.0 (1.0)	+1.55 (-1.25-4.34)	1.11	.27
		Completers	69.3 (19.2)	76.1 (15.6)	70.2 (19.1)	78.1 (16.1)	+1.16 (-2.39-4.71)	0.64	.52
	**Social functioning**								
		Intention-to-treat	79.5 (24.0)	84.0 (1.3)	80.7 (23.0)	86.4 (1.2)	+2.28 (-1.48- 6.04)	0.22	.23
		Completers	79.5 (24.0)	84.4 (20.4)	80.7 (23.0)	87.2 (18.4)	+3.10 (-1.81-8.01)	1.24	.22
	**Bodily pain**								
		Intention-to-treat	79.1 (20.7)	78.9 (1.2)	80.6 (20.0)	79.4 (1.5)	+0.32 (-3.29-3.92)	0.18	.86
		Completers	79.1 (20.7)	79.4 (19.7)	80.6 (20.0)	79.9 (19.0)	+0.18 (-4.82-5.19)	0.07	.94
	**General health perceptions**								
		Intention-to-treat	62.7 (20.1)	66.6 (1.1)	64.3 (20.4)	69.5 (1.3)	+2.64 (-1.10-6.38)	1.42	.16
		Completers	62.7 (20.1)	67.4 (17.0)	64.3 (20.4)	69.7 (18.1)	+1.60 (-1.79-4.99)	0.93	.35
	**Body mass index (kg/m^2^)**^f^								
		Intention-to-treat	28.6 (6.4)	28.3 (0.2)	28.8 (6.5)	27.3 (0.3)	-1.03 (-1.65-0.41)	3.33	.001
		Completers	28.6 (6.4)	26.8 (5.6)	28.8 (6.5)	27.2 (4.5)	-0.58 (-0.95-0.21)	3.08	.002

^a^*t* values are presented for imputed data; Z values (Wald chi-square test: Z=sqrt[chisq]) are presented for completers data.

^b^Statistical significance is represented by *P*<.05.

^c^Standard error (SE) of the mean values are presented for imputed data (intention-to-treat); standard deviation (SD) values are presented for completers data.

^d^In addition to controlling for baseline physical activity, the analyses were adjusted for gender, age, body mass index, and education.

^e^In addition to controlling for the baseline value of the outcome variable, the analyses were adjusted for baseline physical activity, gender, age, body mass index, and education.

^f^In addition to controlling for baseline body mass index, the analyses were adjusted for baseline physical activity, gender, age, and education.

### Website Engagement, Retention, and Usability

**Figure 2:**
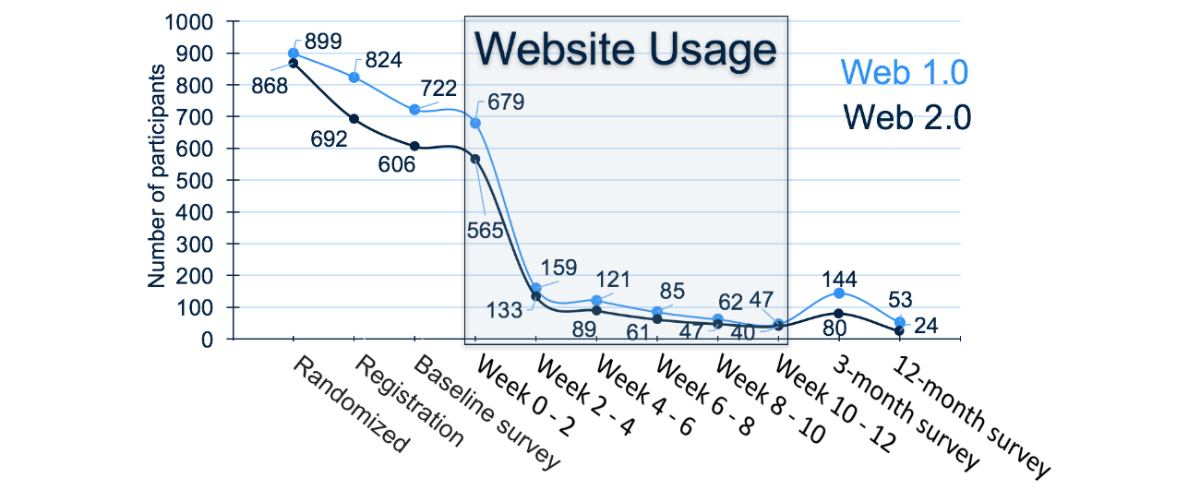
Number of participants engaged with the study and intervention at different time points.

Differences in terms of website usage are shown in Table 3. Participants used the websites on average 3 minutes per week and logged steps for approximately 10 days. There were no significant between-group differences with regard to time on site, entering steps without comments, and the SUS. However, total number of visits (9.0 [19.3] vs 5.7 [17.9], *P*=.002) and average number of visits (0.7 [1.6] vs 0.4 [1.4], *P*=.002) were significantly higher in the Web 2.0 group, as well as the time between first and last visit (3.6 [3.7] vs 2.2 [2.9], *P*<.001). Total days (0.7 [5.9] vs 0.2 [1.5], *P*=.03) and average days (0.05 [0.45] vs 0.01 [0.10], *P*=.02) with a step entry comment were also significantly higher in the Web 2.0 group. A Kaplan-Meier survival plot shows the proportion of participants that remained using the website for each week of the study (see Figure 2). Only 21.99% (292/1328) of participants were still using either website after 2 weeks (n=292 summed for both groups) and 6.55% (87/1328) after 10 weeks. The between-group difference in time-to-nonusage attrition was not statistically significant (Hazard Ratio=0.97, 95% CI 0.86-1.09; *P*=.57). Self-reported use and usefulness of the features available on both websites are reported in Table 4. More participants in the Web 2.0 group used features that were present, or similar, on both websites compared to the Web 1.0 group. Web 2.0 participants also rated the usefulness of these features more highly compared to Web 1.0 participants. Many participants in both groups did not use some of the interactive features or indicate that they were very useful.

**Table 3:** Website engagement, retention, and usability between weeks 1 and 12.

Engagement, retention, and usability metrics	Web 1.0: 10,000 Steps (n=565), mean (SD)	Web 2.0: WALK 2.0 (n=697), mean (SD)	*t*	*P* value^a^
Time on site (seconds per week)	195 (464)	179 (678)	-0.482	.63
Total number of visits	5.71 (17.95)	9.02 (19.34)	3.140	.002
Average number of visits per week	0.46 (1.48)	0.73 (1.58)	3.096	.002
Time between the first and last visit (weeks)	2.22 (2.92)	3.57 (3.75)	7.187	<.001
Total number of days with a step entry	9.97 (21.44)	9.10 (20.88)	-0.731	.47
Average number of days with a step entry per week	0.78 (1.71)	0.71 (1.67)	-0.693	.49
Time between the first and last step entry (weeks)	1.72 (3.43)	1.74 (3.43)	0.070	.95
Total number of days with a step entry comment	0.19 (1.46)	0.70 (5.99)	2.169	.03
Average number of days with a step entry comment per week	0.01 (0.10)	0.05 (0.45)	2.256	.02
Average time for nonusage attrition to occur (weeks)	1.55 (3.16)	1.54 (3.11)	-0.530	.96
Systems Usability Score	61.73 (10.76)	62.53 (11.12)	0.520	.60

^a^Statistical significance is represented by *P*<.05.

**Table 4:** Self-reported use and usefulness of the features available on both websites.

Website features^a^	Web 1.0 (n=144)		Web 2.0 (n=80)	
	Did not use, n (%)	Useful or very useful, n (%)	Did not use, n (%)	Useful or very useful, n (%)
Step entry tool	16 (14.5)	91 (63.1)	6 (8)	63 (79)
Ability to view your step progress	22 (15.2)	89 (61.6)	7 (9)	66 (83)
Articles in the library	51 (35.5)	35 (24.6)	24 (30)	22 (28)
Ability to set goals	32 (22.5)	59 (41.3)	14 (17)	48 (61)
Ability to have and view progress of walking buddies (Web 1.0)/friends (Web 2.0)	58 (40.6)	29 (20.3)	29 (37)	20 (25)
Discussion forum	69 (47.8)	14 (9.4)	29 (37)	13 (16)
Group-based challenges	65 (45.7)	17 (11.6)	21 (26)	17 (21)
Monthly individual challenges	45 (31.2)	42 (29.0)	N/A^b^	N/A
Ability to share your story	72 (50.0)	10 (7.2)	N/A	N/A
Ability to read others’ stories	67 (46.4)	23 (15.9)	N/A	N/A
Ability to like and comment on friends’ updates	N/A	N/A	32 (40)	18 (22)
Ability to send messages to other users	N/A	N/A	33 (41)	17 (21)
Profile page to provide updates	N/A	N/A	31 (38)	19 (24)
Ability to have your own walking blog	N/A	N/A	36 (45)	15 (18)
Ability to read friends’ blog posts	N/A	N/A	31 (38)	19 (24)
Google Maps walking tool	N/A	N/A	40 (50)	15 (18)
Ability to connect with Facebook	N/A	N/A	38 (47)	12 (15)

^a^Some features were present on only one of the two intervention websites; hence, no data are available for those features on the other website.

^b^N/A: not applicable.

## Discussion

### Principal Findings

The aim of this study was to compare physical activity behavior of individuals using a traditional Web 1.0 physical activity website with those using a more interactive and social Web 2.0 physical activity website in real-world settings. The primary hypothesis was confirmed: those in the Web 2.0 group did display significantly higher levels of physical activity at 3 months compared to those in the Web 1.0 group. The secondary hypotheses were partially confirmed: BMI significantly decreased, engagement was significantly higher in some variables (eg, website visits), and quality of life significantly improved in one variable (ie, physical functioning) in the Web 2.0 group compared to the Web 1.0 group at 3 months. However, there were no between-group differences for several other engagement variables (eg, logging steps and nonusage attrition) and most quality-of-life variables. This study was the first to demonstrate the importance of using Web 2.0 features in Web-based physical activity interventions in a real-life setting. These outcomes are strengthened by not finding between-group differences in website usability (ie, SUS score), indicating that outcomes were not influenced by factors such as *user friendliness*. Interestingly, the use and usability of features that were present, or similar, on both websites were higher in the Web 2.0 website compared to the Web 1.0 website. While, from this research, it is not possible to explain this difference given the similar SUS scores, it may in part explain why the Web 2.0 website performed better on several behavioral and engagement outcomes.

While there is an abundance of studies examining the effectiveness of physical activity promotion websites in controlled conditions [33], few studies have examined the use and effectiveness of physical activity websites in natural and real-life conditions. To our knowledge, only one other study has examined people who spontaneously signed up to an online physical activity intervention and where researchers had no direct contact with study participants: all processes were completed automatically [34]. Wanner et al conducted an RCT with two groups of participants that were actively recruited to be randomized to either a control group or an interactive, computer-tailored, physical activity website; however, they included a third group of spontaneous users from the same website. Significantly larger increases in physical activity were observed in the spontaneous users when compared to the actively recruited groups [34]. That study’s outcomes were similar to ours when comparing findings from our RCT with the ecological trial: physical activity increases reported in this ecological trial (+140 for Web 2.0 group; +40 for Web 1.0 group) are much higher than those observed in the RCT (+45.5 for Web 2.0 group; –1.0 for Web 1.0 group) [11]. The large differences observed in these studies indicate that it is not adequate to rely only on RCT outcomes when preparing Web-based physical activity interventions for dissemination and translation.

Poor engagement with Web-based interventions has often been reported; with regard to that aspect, this study is not unique [33,35]. The ecological trial and the RCT [11], however, were remarkably different in terms of engagement and retention outcomes. While the Web 2.0 group also had better engagement outcomes in the RCT, the overall engagement of participants was much lower in the ecological trial. For example, nonusage attrition in the RCT occurred on average after 35.5 and 25.5 weeks in the Web 2.0 and Web 1.0 groups, respectively, whereas it occurred after a mere 1.5 weeks for both groups in the ecological trial. The RCT website was visited, on average, 3.6 and 1.6 times per week for the Web 2.0 and Web 1.0 groups, respectively, but only 0.7 and 0.5 times per week, respectively, in the ecological trial. While participants in both trials were exposed to identical websites, there are factors that encourage engagement in RCTs, and factors that discourage engagement in ecological trials. In the RCT, participants were required to complete multiple face-to-face visits and they received phone calls, a pedometer, and multiple small financial incentives [11]. These strategies increased participant accountability and resulted in a 60% retention rate at 18 months, which is comparable to other studies in this field [6]. In order to keep the intervention implementation as natural as possible in the ecological trial, no such strategies, other than email reminders, were applied. However, in the ecological trial the process of asking 10,000 Steps visitors to participate in research and obtaining informed consent and baseline assessments may have deterred many participants, given the attrition at each step of the process (shown in Figure 1). These necessary steps may have been detrimental to their ongoing participation, as is demonstrated in a previous analysis of 16,948 spontaneous 10,000 Steps users who did not need to go through multiple screenings to be included in the analyses [18]. Among these 10,000 Steps users, nonusage attrition occurred after 4 weeks and the website was visited 2.4 times per week; these engagement outcomes are remarkably higher than those among ecological trial participants [18]. Finally, in another study, Wanner et al compared spontaneous users with trial participants and also reported large engagement differences: nonusage attrition occurred at 41 weeks in trial participants, but it was zero days in spontaneous users [36]. Collectively, these outcomes indicate that caution should be taken when interpreting engagement outcomes from both RCTs and ecological trials, as neither may be a good reflection of how participants engage with websites in reality. The outcomes further suggest that improved participant retention strategies are required that work well in ecologically valid circumstances [37].

The significant reduction of BMI in favor of the Web 2.0 group was surprising, as the intervention did not focus on weight loss, and the 3-month time frame is reasonably short. However, previous Web-based interventions have demonstrated weight loss, both in interventions focused on either weight loss or physical activity [38,39]. Further, few studies have examined how Web-based physical activity interventions can improve health-related quality of life. A meta-analysis of the impact of non-Web-based physical activity interventions on quality of life observed that the interventions only significantly changed the physical functioning domain of quality of life, and not other domains [40]. This is consistent with the outcomes of this study.

Women dominated participation in this study (82%), however, this was very comparable to other studies. For example, Anderson-Bill et al [41] conducted an online walking program and reported that 83% of participants were female and 75% of spontaneous website users were female in the study by Wanner et al [36]. Lower male participation is also commonly observed in RCTs [5,6] and this study demonstrates that the difficulty in attracting men to health behavior change interventions may even be greater in ecologically valid circumstances. Several significant differences were observed between those who participated at 3 months and those who did not. There was less attrition among those randomized to the Web 1.0 group (10,000 Steps). As all participants were originally signing up to participate in the 10,000 Steps program, it is plausible that many of those who were randomized to the Web 2.0 group (WALK 2.0) were disappointed with their allocation and dropped out for that reason. It is also possible that the increased complexity and interactivity of the Web 2.0 website resulted in higher dropout rates, though this is less likely as there was no significant difference in the SUS score between the websites. Older participants and men were less likely to drop out; this is similar to the study by Wanner et al, who found that among their spontaneous users, men and those of increasing age were more likely to repeatedly use their website [36]. It is also in line with a Web-based physical activity intervention study that found older participants spent more time on the website and changed behavior more than younger participants [42]. Finally, it is not surprising to see lower dropout rates among those with higher education, higher income, and a professional occupation, as it has been widely reported that people with a higher socioeconomic status are more amenable to participating in health behavior change interventions [43,44].

### Strengths and Limitations

The major strengths of this study were the innovative ecological randomized nature of the study and the comparison of Web 1.0 and Web 2.0 intervention features. The large nonusage and study attrition, however, was an important limitation, making it problematic to analyze 12-month outcomes. This limitation, though, could be considered as a finding that is of interest, as it is a reflection of how Web-based interventions are being used in ecologically valid circumstances; it is not, per se, a reflection of poor study methodology. That said, several methodological limitations are inherent to ecological trials, such as the lack of a true control group and having to resort to less intrusive (ie, self-report) measures to assess outcomes. These methodological concerns can, however, be alleviated by comparing the study outcomes to those of an RCT that applied more rigorous methods [11]. Furthermore, all engagement and retention measures were assessed objectively through the use of website usage statistics. However, it was a limitation of this study that the interventions were only accessible via websites, as the technology landscape has rapidly changed since the study inception and it is now very common to also use mobile phone apps, which are more convenient to access than websites [18].

### Conclusions

Web 2.0 intervention features appear to be more effective in increasing physical activity, decreasing BMI, and improving physical functioning (ie, quality-of-life domain) compared to Web 1.0 features in ecologically valid circumstances. While the social and interactive Web 2.0 features increased engagement compared to a traditional Web 1.0 website, website usage was low regardless, especially when compared to our previous RCT [11]. As such, more research is needed to increase our understanding of how people engage with Web-based interventions, both in controlled and ecologically valid circumstances, and how closely this engagement is related to actual behavior change (ie, what is the dose-response relationship?) [45]. Finally, the findings of this study are remarkable in how different they are from the findings observed in controlled conditions in terms of attrition, website usage, and behavior changes observed. This is important, because information obtained through RCTs may not translate well to real-world conditions. Yet almost all of our knowledge in this field is derived from RCTs and this has a major influence on how Web-based interventions are designed, what interventions are deemed effective, and what interventions are being disseminated and implemented. As such, there is an urgent need for more ecological trials and implementation studies.

## Appendix

Multimedia Appendix 1 Conference presentation slides and screenshots.

Multimedia Appendix 2 CONSORT-EHEALTH checklist (V.1.6.1).

## Abbreviations

ANCOVA: analysis of covariance
BMI: body mass index
N/A: not applicable
RCT: randomized controlled trial
RR: Relative Risk
SUS: System Usability Scale
