# Supplementary material for: Effectiveness of a Web 2.0 Intervention to Increase Physical Activity in Real-World Settings: Randomized Ecological Trial
Source: J Med Internet Res. 2017 Nov 13;19(11):e390. doi: 10.2196/jmir.8484 (PMC5703981; doi:10.2196/jmir.8484)
Supplement: Multimedia Appendix 1 [file jmir_v19i11e390_app1.pptx]

## Slide 1
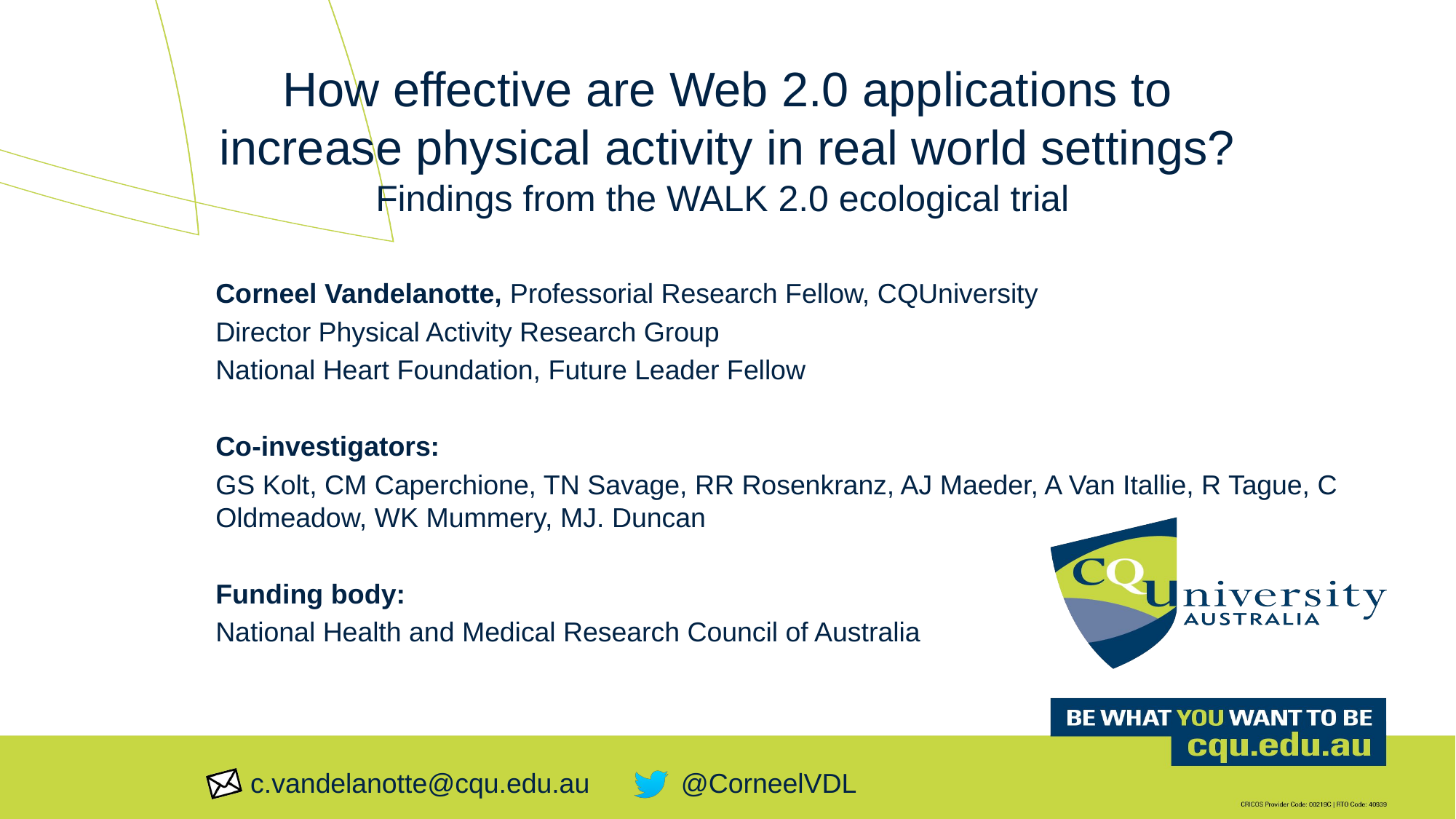

# How effective are Web 2.0 applications to increase physical activity in real world settings? Findings from the WALK 2.0 ecological trial
Corneel Vandelanotte, Professorial Research Fellow, CQUniversity
Director Physical Activity Research Group
National Heart Foundation, Future Leader Fellow
Co-investigators:
GS Kolt, CM Caperchione, TN Savage, RR Rosenkranz, AJ Maeder, A Van Itallie, R Tague, C Oldmeadow, WK Mummery, MJ. Duncan
Funding body:
National Health and Medical Research Council of Australia
c.vandelanotte@cqu.edu.au @CorneelVDL

## Slide 2
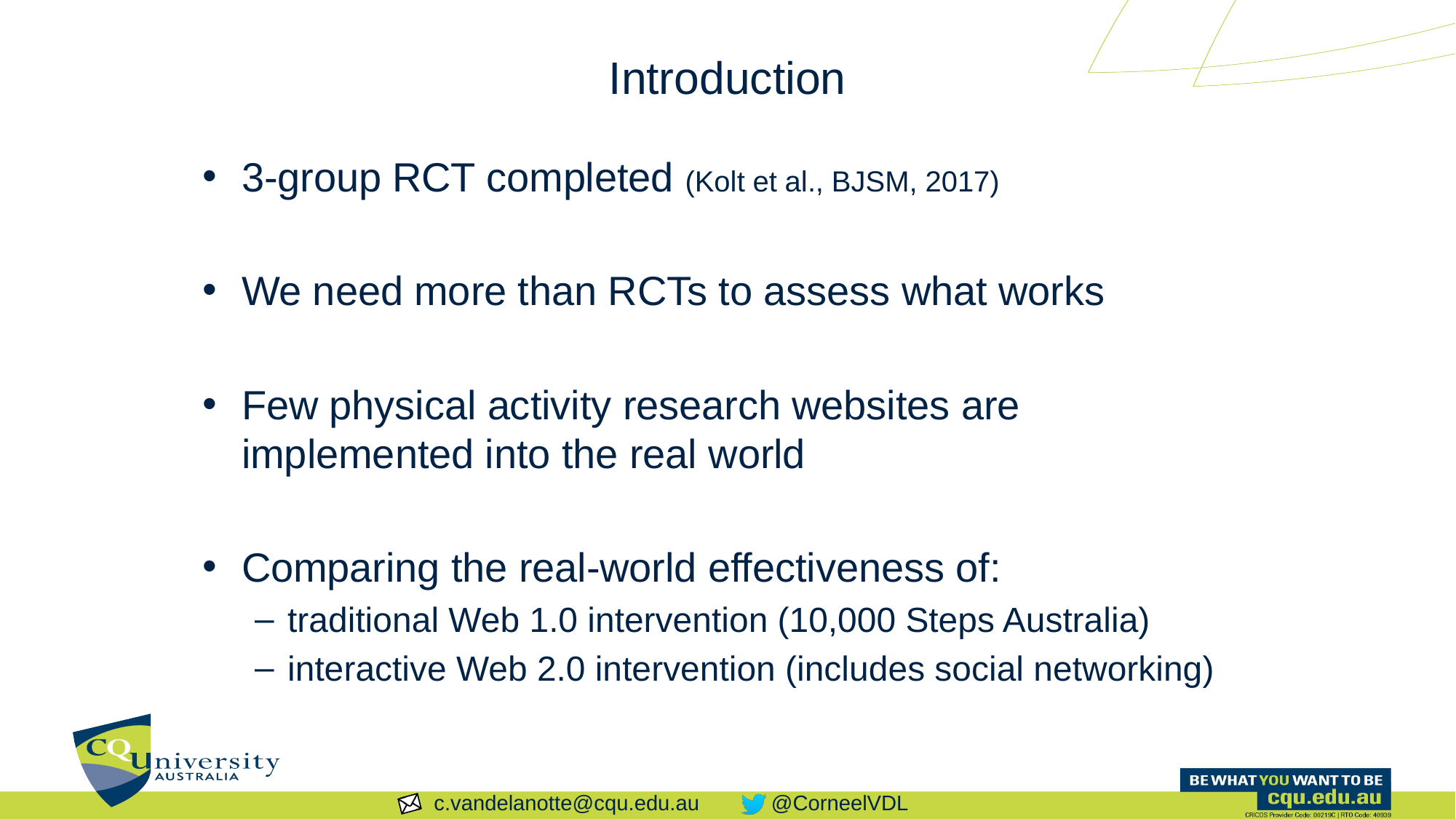

# Introduction
3-group RCT completed (Kolt et al., BJSM, 2017)
We need more than RCTs to assess what works
Few physical activity research websites are implemented into the real world
Comparing the real-world effectiveness of:
traditional Web 1.0 intervention (10,000 Steps Australia)
interactive Web 2.0 intervention (includes social networking)
c.vandelanotte@cqu.edu.au @CorneelVDL

## Slide 3
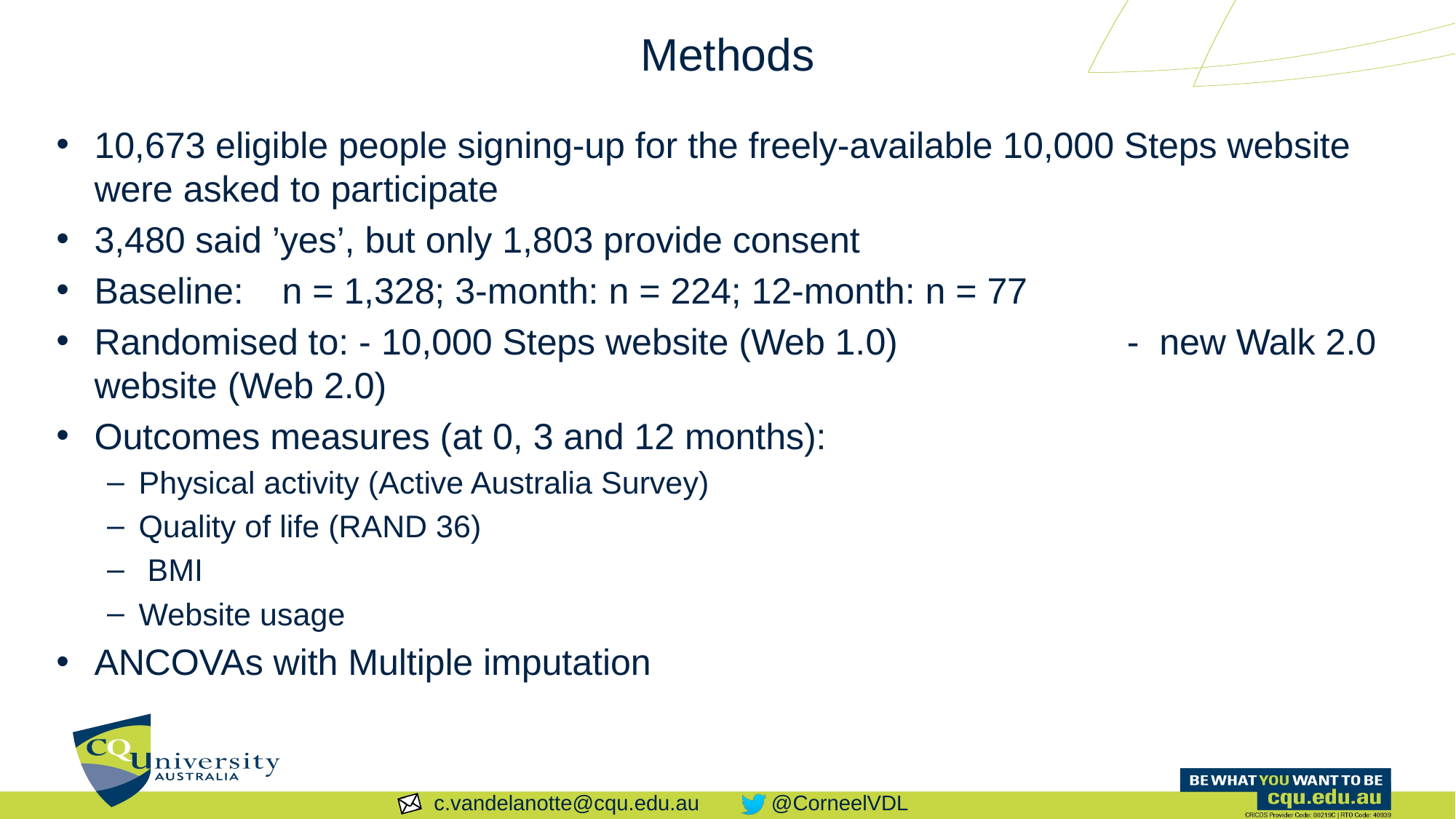

# Methods
10,673 eligible people signing-up for the freely-available 10,000 Steps website were asked to participate
3,480 said ’yes’, but only 1,803 provide consent
Baseline: 	n = 1,328; 3-month: n = 224; 12-month: n = 77
Randomised to: - 10,000 Steps website (Web 1.0) 											- new Walk 2.0 website (Web 2.0)
Outcomes measures (at 0, 3 and 12 months):
Physical activity (Active Australia Survey)
Quality of life (RAND 36)
 BMI
Website usage
ANCOVAs with Multiple imputation
c.vandelanotte@cqu.edu.au @CorneelVDL

## Slide 4
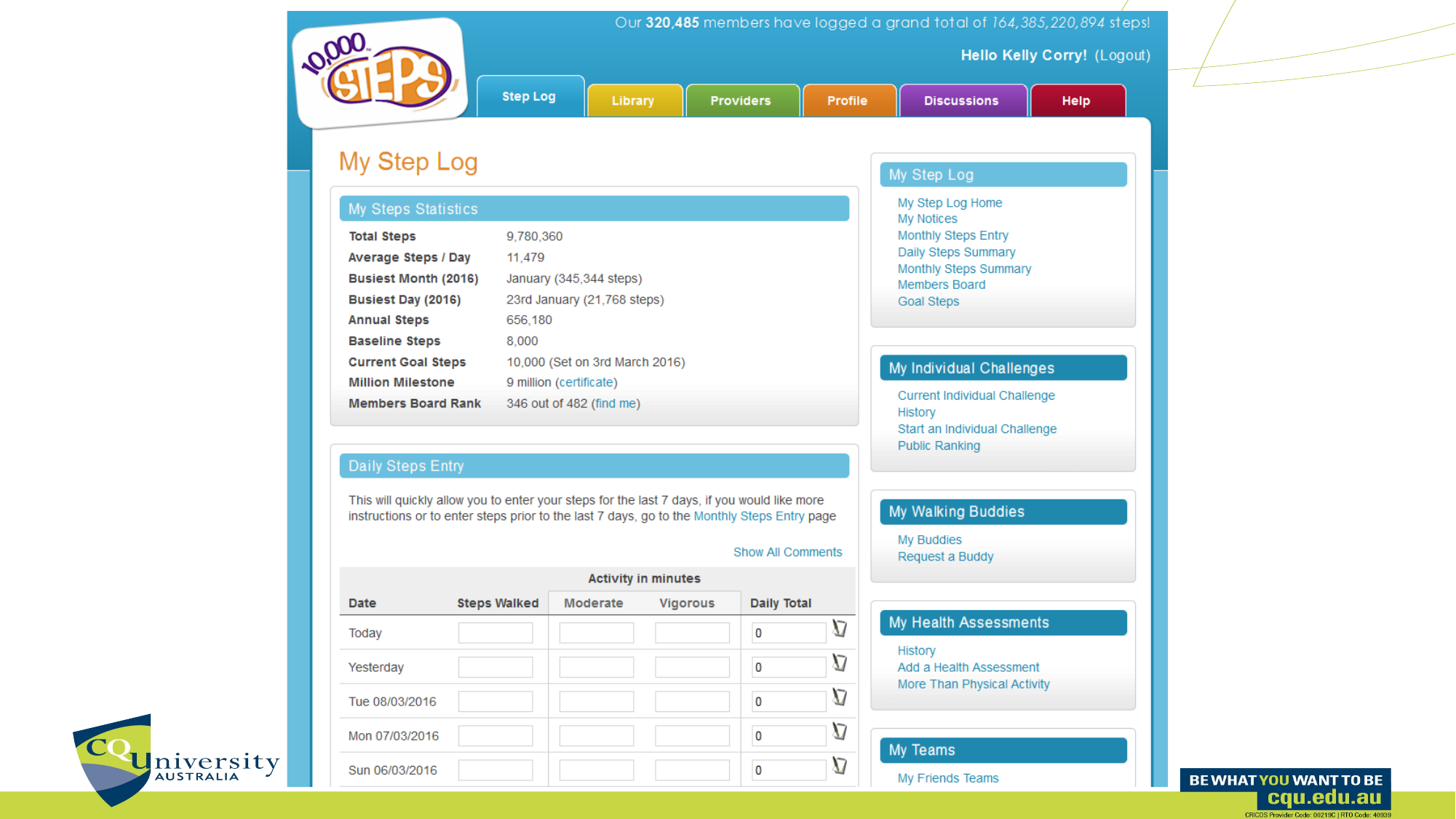

#

## Slide 5
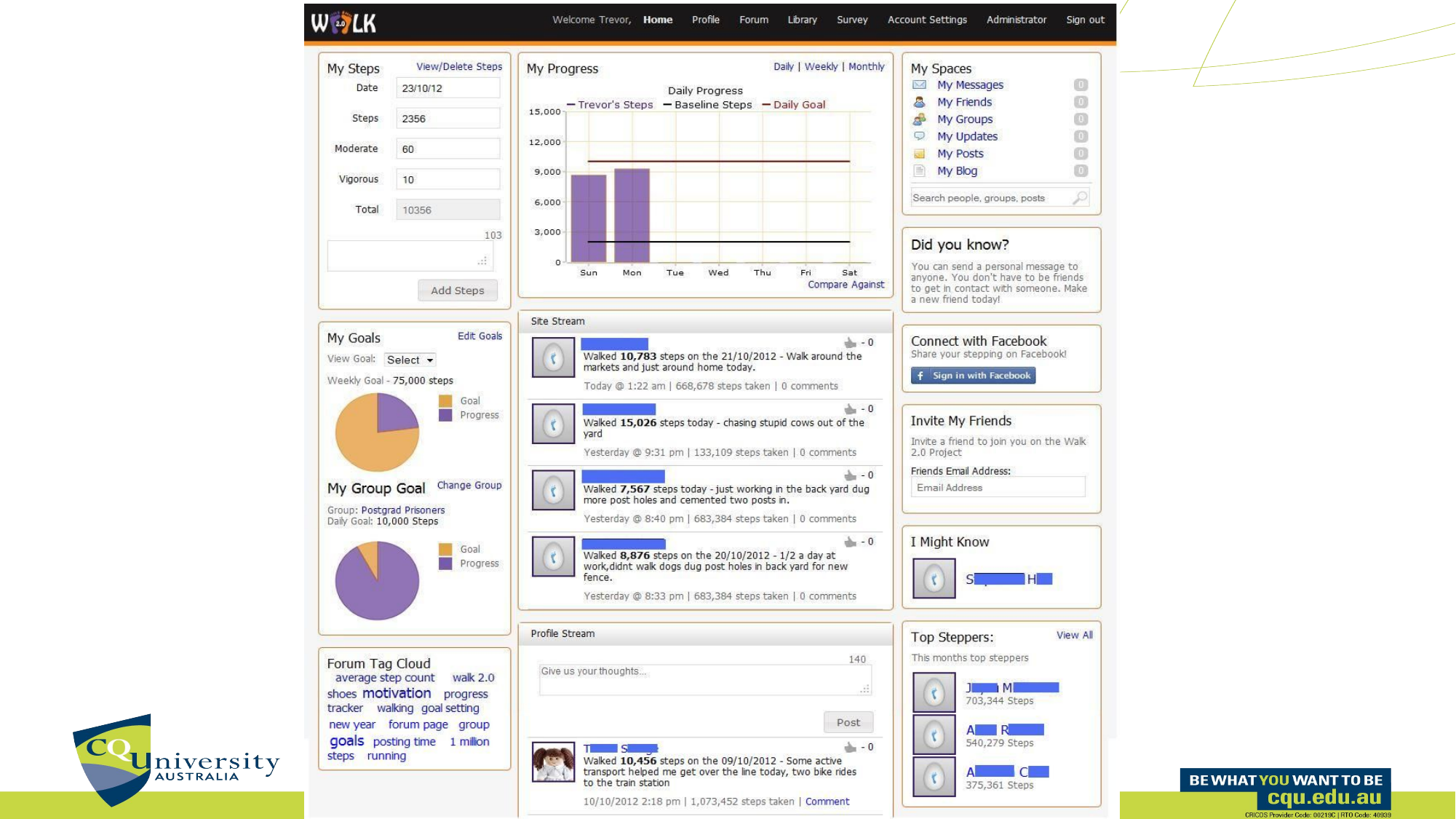

#

## Slide 6
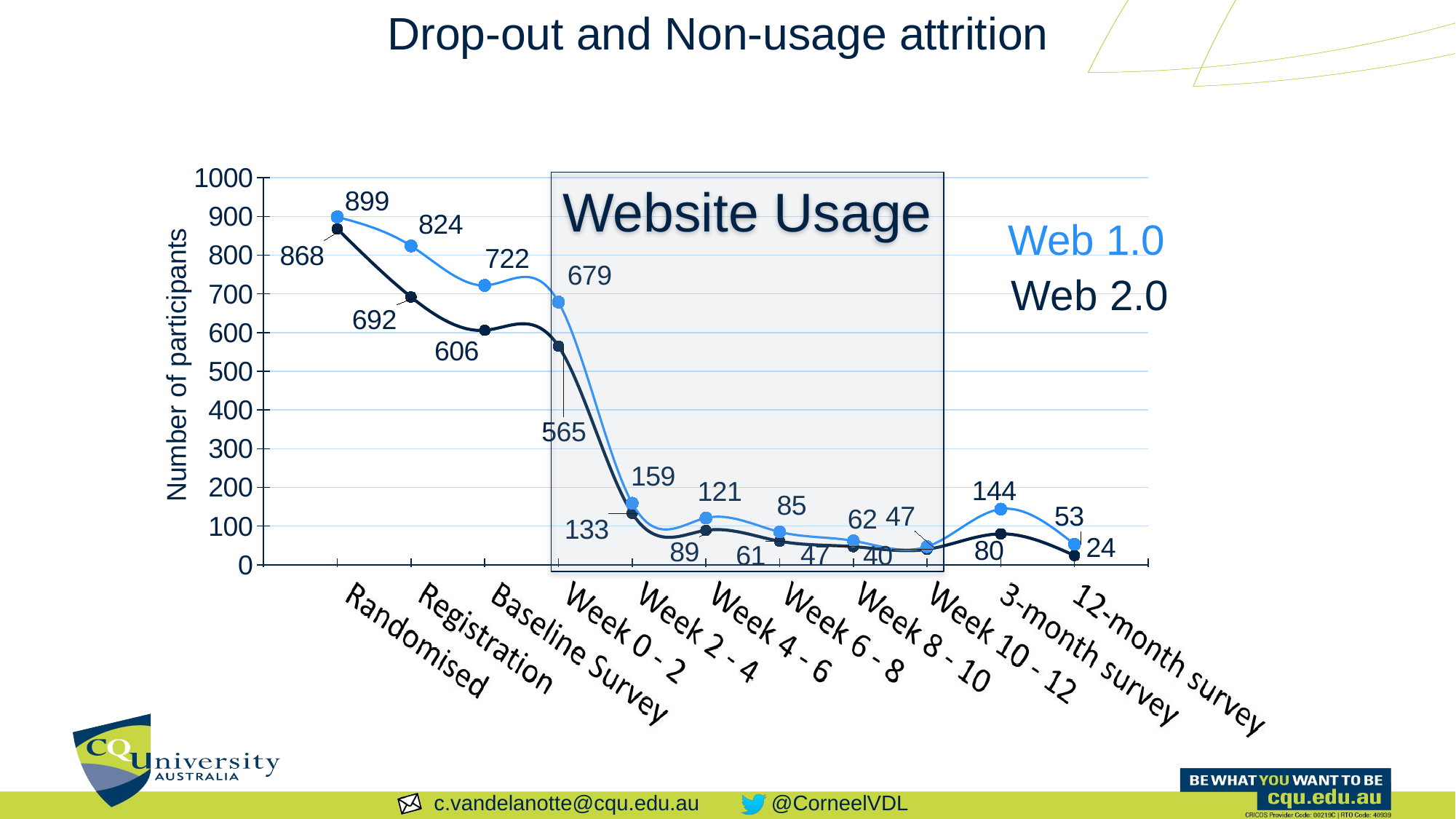

# Drop-out and Non-usage attrition
### Chart
| Category | Web 1.0 | Web 2.0 |
|---|---|---|Website Usage
Web 1.0
Web 2.0
Number of participants
c.vandelanotte@cqu.edu.au @CorneelVDL

## Slide 7
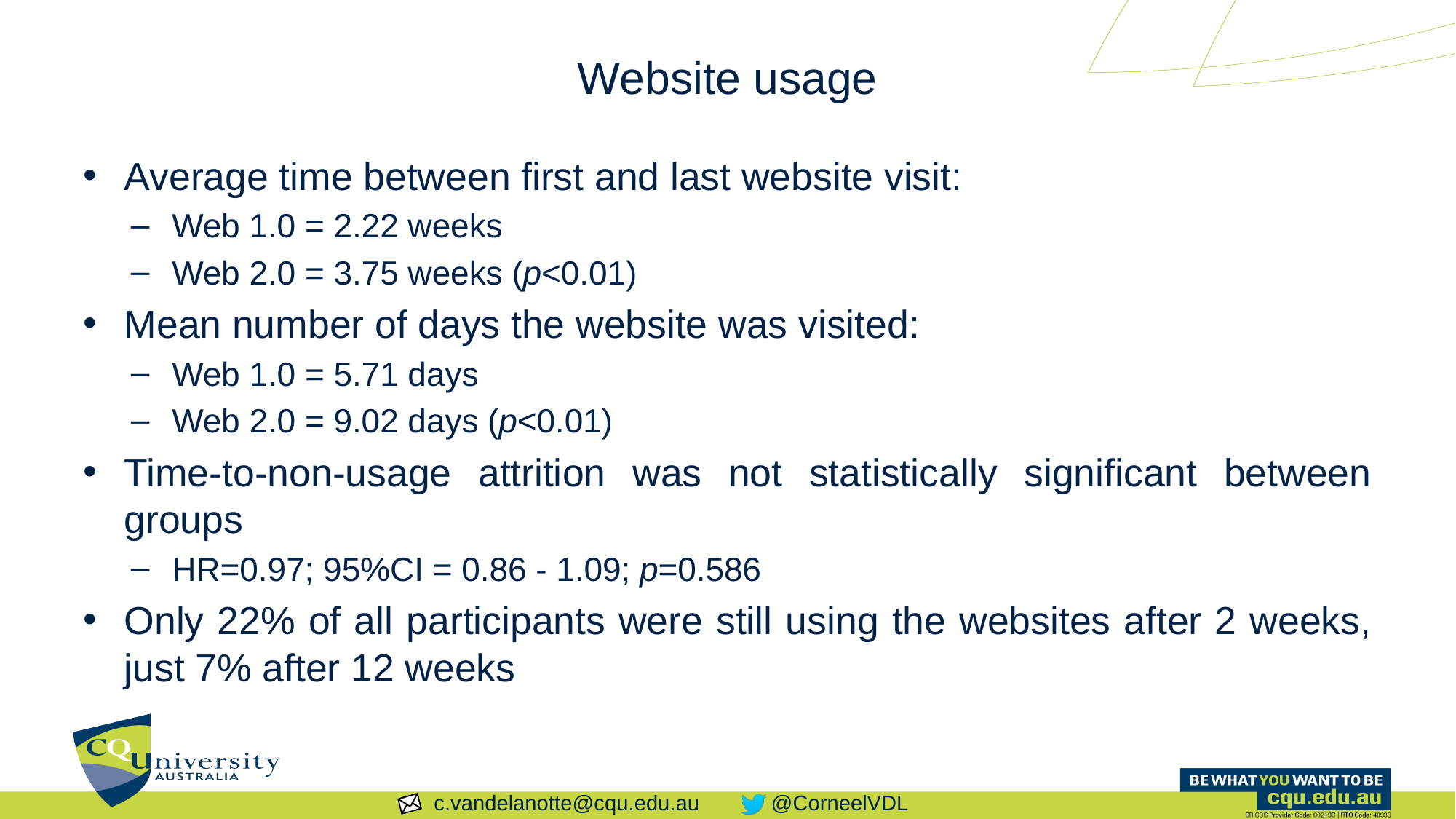

# Website usage
Average time between first and last website visit:
Web 1.0 = 2.22 weeks
Web 2.0 = 3.75 weeks (p<0.01)
Mean number of days the website was visited:
Web 1.0 = 5.71 days
Web 2.0 = 9.02 days (p<0.01)
Time-to-non-usage attrition was not statistically significant between groups
HR=0.97; 95%CI = 0.86 - 1.09; p=0.586
Only 22% of all participants were still using the websites after 2 weeks, just 7% after 12 weeks
c.vandelanotte@cqu.edu.au @CorneelVDL

## Slide 8
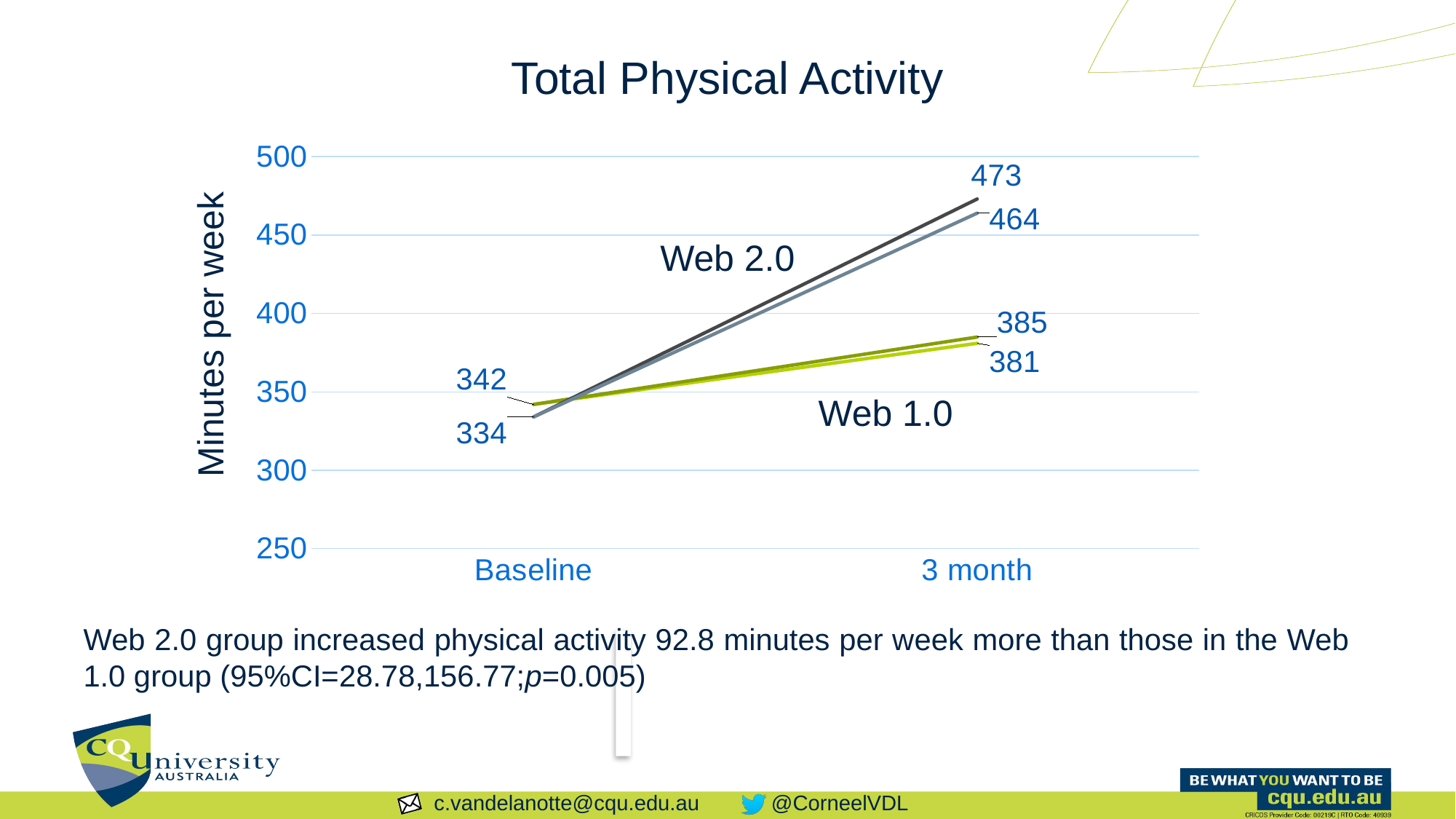

# Total Physical Activity
### Chart
| Category | Web 1.0 ITT | Webi 1.0 CT | Web 2.0 ITT | Web 2.0 CT |
|---|---|---|---|---|
| Baseline | 342.0 | 342.0 | 334.0 | 334.0 |
| 3 month | 381.0 | 385.0 | 473.0 | 464.0 |Web 2.0
Minutes per week
Web 1.0
Web 2.0 group increased physical activity 92.8 minutes per week more than those in the Web 1.0 group (95%CI=28.78,156.77;p=0.005)
c.vandelanotte@cqu.edu.au @CorneelVDL

## Slide 9
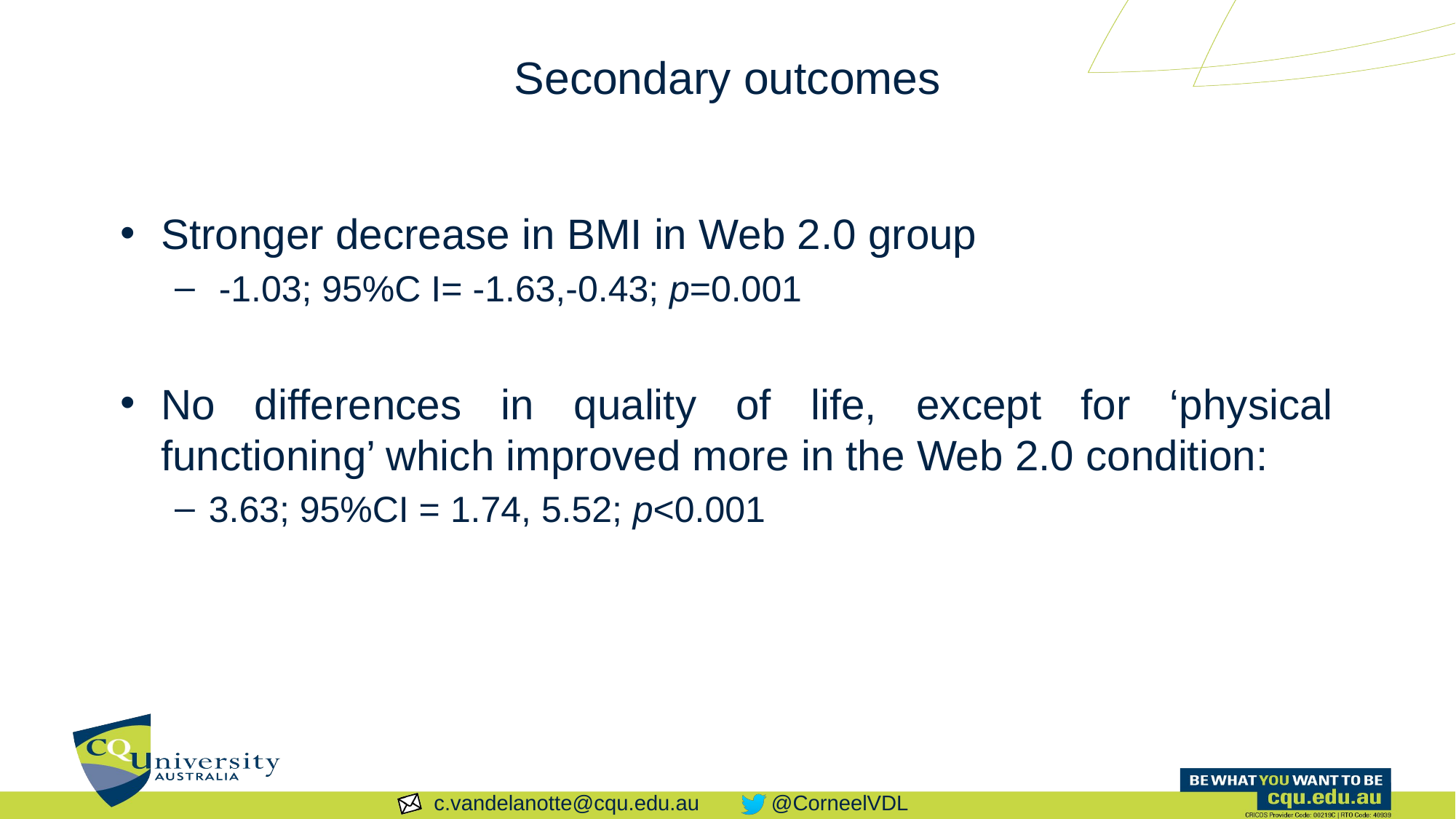

# Secondary outcomes
Stronger decrease in BMI in Web 2.0 group
 -1.03; 95%C I= -1.63,-0.43; p=0.001
No differences in quality of life, except for ‘physical functioning’ which improved more in the Web 2.0 condition:
3.63; 95%CI = 1.74, 5.52; p<0.001
c.vandelanotte@cqu.edu.au @CorneelVDL

## Slide 10
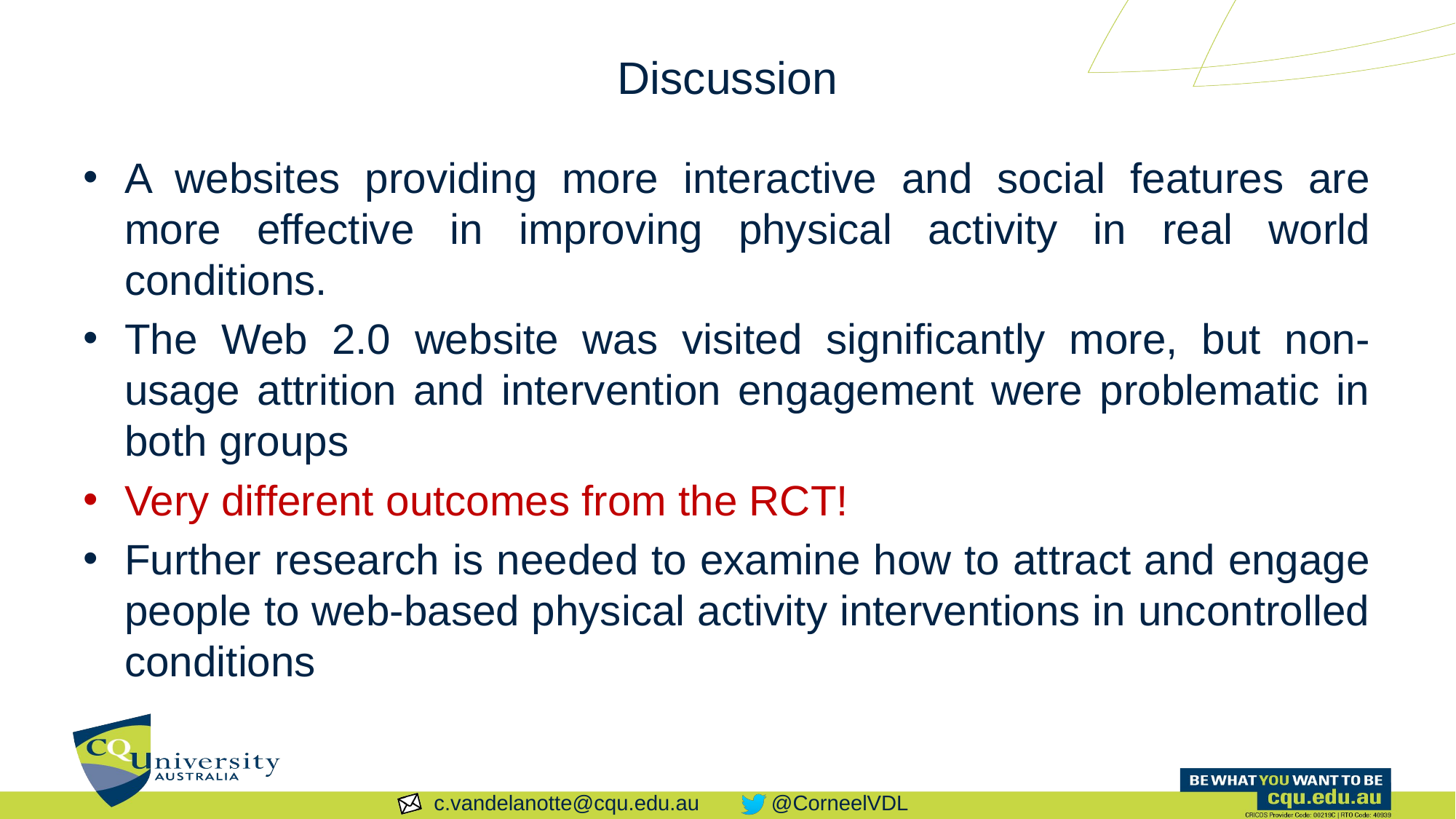

# Discussion
A websites providing more interactive and social features are more effective in improving physical activity in real world conditions.
The Web 2.0 website was visited significantly more, but non-usage attrition and intervention engagement were problematic in both groups
Very different outcomes from the RCT!
Further research is needed to examine how to attract and engage people to web-based physical activity interventions in uncontrolled conditions
c.vandelanotte@cqu.edu.au @CorneelVDL
